# Periodontal Reasons for Tooth Extraction in a Group of Greek Army Personnel

**DOI:** 10.5681/joddd.2011.012

**Published:** 2011-06-14

**Authors:** Nikolaos Andreas Chrysanthakopoulos

**Affiliations:** Post-graduate Student, Department of Maxillofacial and Oral Surgery, 401 General Military Hospital of Athens, Athens, Greece

**Keywords:** Non-commissioned officers, officers, periodontal disease, permanent teeth, tooth extraction

## Abstract

**Background and aims:**

The aim of this study was to investigate the prevalence of permanent teeth extracted due to periodontal disease and its relation to age, military rank, and type of extracted teeth due to periodontal and non-periodontal reasons among a group of Greek Army personnel attending a military dental practice.

**Materials and methods:**

Study population consisted of 509 officers, non-commissioned officers and soldiers, aged 18 to 44 years from a military dental hospital in Greece. The reasons for extractions of teeth for a period of two years were obtained, including aspects such as age, military rank and the type of teeth extracted due to periodontal and non-periodontal reasons. Data were analyzed using chi-squared test.

**Results:**

The total number of extracted teeth was 1,231, of which 34.4% were extracted because of periodontal reasons, 32.2% for dental caries and 33.4% for other reasons. The average number of extracted teeth due to periodontal disease showed an increase with age. Maxillary and mandibular first and second molars were the most frequently extracted teeth due to periodontal reasons; however, the anterior teeth of both jaws with mobility (grade III), the same teeth with attach-ment loss (≥5.0 mm) and the posterior teeth of both jaws with furcation involvement (grade IV) were the most frequently extracted teeth due to periodontal reasons.

**Conclusion:**

Although the goal of the WHO regarding the reduction of dental caries was accomplished, periodontal dis-ease was still the main cause of tooth extraction and showed an increase with age.

## Introduction

Tooth extraction, regardless of the progress of modern dentistry causes serious problems and dysfunction of the masticatory system and is thought to be a complex problem for both the clinical dentist and the patient.^1^ In addition, the number of extracted teeth might serve as an indicator of the socio-economic and the oral hygiene level. Decrease in the number of teeth results in poor dietary habits and deterioration of quality of life.^2^ Therefore, it is important to investigate the reasons for permanent tooth extraction. Based on this information the community dentistry could put into effect adequate dental health policies.^3^

The main reasons for tooth extraction, in general, are: dental caries, periodontal disease, the combination of dental caries and periodontal disease, accident injuries, orthodontic reasons, impacted teeth (e.g. canines, lateral incisors), failed dental treatments (root canal treatments, etc.).^4-6^ The philosophy of modern dentistry focuses its efforts on the maintenance of permanent teeth putting into practice several preventive dentistry programs for the whole population.^3^

Previous studies have strongly implicated dental caries and periodontal disease as the major causes of tooth extraction in several countries. In those studies dental caries appeared to be the main cause of tooth extraction in a large number of countries^5,7-20^, and the number of extracted teeth showed an increase with age. Only four studies have shown that the main reason for tooth extraction, regardless of age, is periodontal disease.^6,21-23^

The aim of the present study was to estimate the prevalence of permanent teeth extracted due to periodontal disease and to investigate its correlation with age, military rank, and type of the teeth extracted due to periodontal and non-periodontal reasons.

## Materials and Methods

### Subjects

Study population consisted of 509 officers, non-commissioned officers and soldiers (491 males, 18 females), 18-44 years of age (mean age of 25.3±4.5). The present study was carried out in a military dental hospital in Komotini, a border town in south-eastern Greece. The reasons for tooth extractions were obtained in the subjects for a period of two years, which included variables such as age, military rank and the type of teeth extracted due to periodontal and non-periodontal reasons.

A comprehensive history was taken and all the examinations, clinical measurements and extractions were performed by the author of the present study.

The subjects were divided into three groups according to age range: group I: 18 to 24 years, 122 patients; group II: 25 to 34 years, 190 patients; group III: 35 to 44 years, 197 patients. The participants were in good general health as estimated by a health questionnaire.

### Ethical considerations

The present study was not an experimental one. In Greece only experimental studies must be reviewed and approved by authorized committees (Dental Schools, Greek Dental Associations, Ministry of Health, etc.). Subjects who agreed to participate in the present study were informed about the evaluation to which they would be subjected and signed an informed consent form.

Patients with diagnosed pathological conditions were advised to seek consultation and treatment.

### Clinical examination

The clinical measurements of the participants were performed by the author of the present study as mentioned above.

Tooth mobility, severity of attachment loss and furcation involvement were the main criteria, which indicate the extraction of periodontally affected teeth.^24,25^

The teeth and gingiva were dried with compressed air while dental unit light was used as the light source for the inspections.

The following measurements were carried out on each tooth with poor prognosis:

- The distance in millimetres from cementoenamel junction (CEJ) to the bottom of the gingival sulcus using a William’s probe (Goldman–Fox/Williams Color-Coded Probe PCPGF/W) in the mid-facial buccal surfaces of all the teeth except for the third molars in order to estimate the attachment loss; in cases in which the CEJ was covered by calculus, hidden by a restoration or lost due to caries or wear lesions the location of such junction was estimated on the basis of the adjacent teeth. Periodontal destruction was considered severe when 5.0 mm or more of clinical attachment loss had occurred.
- The clinical grade of mobility: Each tooth was held firmly between the handles of two metallic instruments and an effort was made to move it in all directions. Only grade III mobility (severe mobility faciolingually and/or mesiodistally, combined with vertical displacement) was recorded.
- The clinical grade of furcation involvement according to Glickman’s classification (grades I-IV): Only grade IV furcation involvement, in which the periodontal probe passed readily from one aspect of the tooth to another, was recorded. Evaluation of furcation involvement was performed by using a Nabers probe (2N Nabers DE Probe: P2N). Other reasons for tooth extraction were the following: dental caries and its sequela (fracture of teeth weakened by caries or endodontics), periodontal reasons and dental caries, root trauma, vertical fracture of crown/root, orthodontic reasons, and failed dental treatments.

### Inclusion criteria

The selection criteria comprised age above 18 years and a mean number of 20 natural teeth, since large numbers of missing teeth might have interfered with the results of the present study.

### Exclusion criteria

Third molars were excluded from the study. None of the participants had received scaling and root planing or periodontal treatment during the previous six months.

### Statistical analysis

The statistical unit of the present study was the tooth. For each patient average values of variables such as tooth mobility (M), loss of attachment (LAT) and furcation involvement (FINV) were calculated. As appropriate, chi-squared test was employed to test the hypothesis of no differences between officers, non-commissioned officers and soldiers regarding the average number of extracted teeth overall, the average number of extracted teeth due to periodontal and non-periodontal reasons and the number of extracted teeth due to periodontal reasons. Data analysis was performed using the statistical package of SPSS Ver.16.0 program package (SPSS Inc., Chicago, IL). Statistical significance was defined at P < 0.05.

## Results

A total of 1231 permanent teeth, extracted from 509 officers, non-commissioned officers and soldiers, were surveyed.

The composition of the sample of the study according to age group was as follows: 18-24 year-olds: 24% for the soldiers and 24% for the officers and non-commissioned officers; 25-34 year-olds: 35.2% for the soldiers and 40% for the officers and non-commissioned officers; 35-44 year-olds: 40.8% for the soldiers and 36% for the officers and non-commissioned officers.

The distribution (%) of teeth extracted due to periodontal and non-periodontal reasons according to age group by military rank was: 18-24 year-olds: 16% for the soldiers and 9% for the officers and non-commissioned officers; 25-34 year-olds: 27.7% for the soldiers and 27% for the officers and non-commissioned officers; 35-44 year-olds: 56.3% for the soldiers and 64% for the officers and non-commissioned officers.

The average number of missing teeth was overall 2.42, 1.88 in officers and non-commissioned officers and 2.84 in soldiers, with statistically significant differences (P < 0.001); however, the average number of extracted teeth due to periodontal disease was overall 0.83, 0.68 in officer and non-commissioned officers and 0.95 in soldiers, with statistically significant differences (P < 0.001).

**Figure 1:**
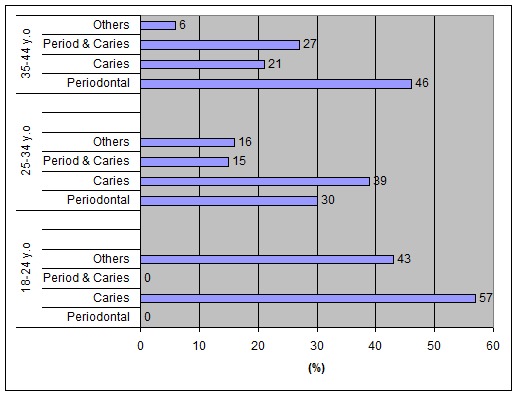
Distribution (%) of teeth extracted in each age group based on the reasons. *Other reasons: root trauma, vertical fracture of crown/root, orthodontic reasons, failed dental treatments.

A similar distribution regarding the average number of teeth extracted due to periodontal and non-periodontal reasons was: 18-24 year-olds: 1.91 for the soldiers and 0.7 for the officers and non-commissioned officers; 25-34 year-olds: 2.24 for the soldiers and 1.27 for the officers and non-commissioned officers; 35-44 year-olds: 3.91 for the soldiers and 3.34 for the officers and non-commissioned officers.Figure 1 shows the distribution (%) of teeth extracted in each age group based on periodontal and non-periodontal reasons. The differences between the teeth extracted due to periodontal reasons and due to other causes between officers, non-commissioned officers and soldiers were not statistically significant.

Regarding the rates (%) of teeth extracted due to periodontal reasons in each age group by military rank, the distribution was as follows: 18-24 year-olds: 0% for the soldiers and 0% for the officers and non-commissioned officers; 25-34 year-olds: 62.6% for the soldiers and 37.4% for the officers and non-commissioned officers; 35-44 year-olds: 64.7% for the soldiers and 35.3% for the officers and non-commissioned officers.

**Figure 2:**
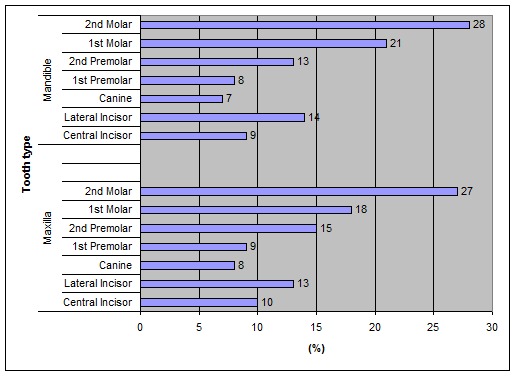
Distribution (%) of extractions due to periodontal reasons by tooth type in the maxilla and mandible.

Figure 2shows the extracted teeth (%) due to periodontal reasons by tooth type.

Generally the first and second molars of both jaws were the more extracted teeth for periodontal reasons followed by the lateral and central incisors.

**Figure 3:**
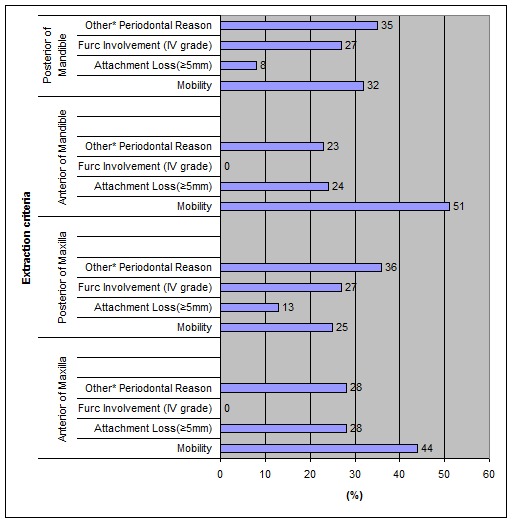
Distribution (%) of teeth extracted due to periodontal reasons solely (extraction criteria by Moreira et al.) *Other periodontal reasons include: radiographic bone loss (RBL) greater than 50% prosthetic planning, socio-economic and cultural aspects (Moreira et al.)

Figure 3 presents the percentages (%) of tooth groups extracted due to periodontal reasons only, according to the criteria determined by Moreira et al.^24^

Upper anterior (44%) and lower anterior (51%) teeth were often extracted due to mobility (grade III).

It was also observed that the anterior teeth of the maxilla (28%) and mandible (24%) were the most frequently extracted teeth due to attachment loss (≥5.0 mm); 28% of the posterior teeth of the maxilla and 27% of the posterior teeth of the mandible were extracted due to furcation involvement (grade IV).

## Discussion

Tooth extraction causes serious problems and dysfunction of the masticatory system. Previous studies have shown that dental caries and periodontal disease are the main causes of tooth extraction in several countries,^5-23,26,27^ and the percentage ranges from 31.8%^20^ to 94.4%.^4^ As mentioned the aim of the present study was to estimate the periodontal reasons for tooth extraction in a Greek Army Personnel.

Moreira et al^24^ showed that tooth mobility (M), severity of attachment loss (LAT) and radiographic bone loss (RBL) greater than 50%, are the most frequently adopted criteria to indicate the extraction of periodontally affected teeth.

Other criteria that have been adopted to indicate the extraction of teeth with periodontitis are prosthetic planning, furcation involvement, socio-economic and cultural aspects that may be related to the wish of patients to undergo certain types of dental treatments.^24^

Another study by Warren et al^28^ showed that by the use of Generalized Estimating Equations (GEE) they identified cost of treatment, presence of tooth mobility, poor prognosis of alternative treatment and presence of gross caries as significant factors associated with extraction while previous treatment of the tooth and concerns with patients’ health were significantly associated with alternatives to extraction.

Bercy & Blase^29^ suggested the following elements of the decision to extract teeth for periodontal reasons: function and strategic importance of the tooth, extension of the lesion, level of inter-radicular attack, endo-periodontal lesions, fractures and luxations, radicular proximity, implication of wisdom teeth and evolution after treatment.

The present study showed that the average number of missing teeth was overall 2.42, which is low compared to similar studies carried out in Greece ^30-32^ during the last decades. This observation shows the improvement of socio-economic level, the interest of the Greek population regarding its oral health, the acceptance of the value and importance of the role of preventive dentistry.

Dental caries appears to be the main cause of tooth extraction in a large number of countries with the following percentages: 70.3%,^4^ 67,5%,^7^ 63.3%,^11^ 59.2%,^13^ 59%,^14^ 56.4%,^15^ 52.6%,^12^ 50.0%,^16^ 51%,^5^ 47.9%,^19^ 46.9%,^17^ 43.7%,^9^ 43.3%,^10^ and 39.5%.^33^

Only two studies have shown that both caries and periodontal disease are almost equally important reasons for tooth extraction, such as in Italy and Japan.^10,27^

Periodontal disease appears to be the main reason for tooth extraction in a small number of previous studies, including the present study in which periodontal disease was the main reason for tooth extraction (34.4%), followed by dental caries (32.2%). A study in an Asian population^23^ found that 35.8% of the extractions were due to periodontal disease and 35.4% due to dental caries. Another study in Germany showed that 27.3% of the extractions were due to periodontal reasons and 20.7% due to dental caries.^22^ The same results were achieved in Canada and Jordan.^6,26^ A high percentage (33.4%) of the other causes such as accident injuries, orthodontic reasons, impacted teeth, failed dental treatments (e.g. root canal treatments) was recorded in the present study.

The above differences might be attributed to the heterogeneous population samples under study, the progression of dental caries and periodontal disease during the last decades, the different methods which were used in order to estimate the frequency of permanent teeth extracted (e.g. clinical examination, questionnaire), and the possible negative attitude of several population samples to seek preventive dental follow-up regularly. The present study concerned subjects who sought dental treatment in a military dental hospital; therefore, the sample could not be considered a random one. On the other hand, no similar studies regarding the periodontal reasons for tooth extraction in an army personnel in Greece have been carried out previously.

In the present study the teeth extracted due to periodontal disease showed a significant increase with age, which is similar to previous studies. ^10,11,17,22,23,34^ However, other studies have shown that periodontal disease is the main cause of extraction in patients up to and over 35 years of age.^9,10,13,16-18,35^ The finding might be attributed to the small population size in the present study and possibly to the negative attitude of the elderly people to seek preventive dental follow-up.

According to the present study the first and second molars of maxilla and mandible were more frequently extracted for periodontal reasons. Previous studies have shown that the more frequently extracted teeth due to periodontal reasons are the molars of the mandible,^15,36^ the molars of both jaws,^19^ maxillary teeth,^36^ the central incisors of the mandible,^17,23,35,37^ the canines and incisors of both jaws, ^9,13^ the anterior teeth of the maxilla^20,35^ and the posterior teeth of both jaws.^12,13^ Those differences could be attributed to the factors mentioned above.

In the present study tooth mobility (M) (grade III), severity of attachment loss (LAT) (≥5.0 mm) and furcation involvement (FINV) (grade IV) were the adopted criteria to indicate the extraction of periodontally affected teeth. Pre-extraction radiographs were not taken in order to use the (RBL) index as a criterion, which indicates the extraction of periodontally affected teeth. In addition, the majority of the participants refused to undergo a radiographic examination.

Under those conditions tooth mobility and furcation involvement were confirmed as the sole cause of tooth mortality according to the findings of previous studies for the posterior teeth of the maxilla and mandible.^5,8,10^ However, tooth mobility and attachment loss were confirmed as the sole cause for tooth mortality according to the findings of other studies for the anterior teeth of both jaws.^2,5,7,10,13,17,18^

According to the results of the present study, periodontal disease and dental caries remain the main causes for extraction of permanent teeth in young and middle-aged people. The role of the clinical dentist is quite important in maintaining the permanent teeth, especially in the middle-aged people who miss their teeth due to periodontal disease.

## Conclusion

1. Periodontal disease and dental caries were the main causes for tooth extraction for officers, non-commissioned officers and soldiers and the frequency of teeth extracted due to periodontal disease increased with age.
2. The more frequently extracted teeth due to periodontal disease were the central and lateral incisors of both jaws; however, the more frequently extracted posterior teeth due to periodontal disease were the first and second molars of both jaws.
3. Anterior teeth of maxilla and mandible were more often extracted teeth due to mobility (grade III); in addition the same teeth were the most frequently extracted teeth due to attachment loss (≥5.0 mm).
4. Posterior teeth of the maxilla and mandible were exclusively extracted due to furcation involvement (grade IV).
